# Influence of Bone-Level Dental Implants Placement and of Cortical Thickness on Osseointegration: In Silico and In Vivo Analyses

**DOI:** 10.3390/jcm11041027

**Published:** 2022-02-16

**Authors:** Javier Gil, Clara Sandino, Miguel Cerrolaza, Román Pérez, Mariano Herrero-Climent, Blanca Rios-Carrasco, Jose Vicente Rios-Santos, Aritza Brizuela

**Affiliations:** 1 Faculty of Medicine and Health Sciences, Bioengineering Institute of Technology, International University of Cataluña, c. Josep Trueta s/n, 08125 Sant Cugat del Valles, Barcelona, Spain; mcerrolaza@uic.es (M.C.); rperezan@uic.es (R.P.); 2 Faculty of Denstistry, International University of Cataluña, c. Josep Trueta s/n, 08125 Sant Cugat del Valles, Barcelona, Spain; 3 Facultat de Ciències i Tecnologia, Universitat de Vic-Central de Catalunya, C/de la Laura, 13, 08500 Vic, Barcelona, Spain; claraines.sandino@uvic.cat; 4 Porto Dental Institute, 4150-518 Porto, Portugal; dr.herrero@herrerocliment.com; 5 Department of Periodontology, Dental School, University of Seville, 41009 Seville, Seville, Spain; brios@us.es (B.R.-C.); jvrios@us.es (J.V.R.-S.); 6 Departamento de Cirugía y Especialidades Médico-Quirúrgicas, Universidad de Oviedo, 33006 Oviedo, Asturias, Spain; aritzabrizuela@hotmail.com

**Keywords:** osseointegration, bone, dental implants, cortical bone, in vivo, histology

## Abstract

The purpose of this research is to study the biomechanical response of dental implants in bone-level type locations, 0.5 mm above and below the bone level. In addition, the influence of the thickness of the cortical bone on osseointegration is determined due to the mechanical loads transfer from the dental implant to the cortical and trabecular bone. The thicknesses studied were 1.5 mm and 2.5 mm. Numerical simulations were performed using a finite element method (FEM)-based model. In order to verify the FEM model, the in silico results were compared with the results obtained from a histological analysis performed in an in vivo study with 30 New Zealand rabbits. FEM was performed using a computerized 3D model of bone-level dental implants inserted in the lower jawbone with an applied axial load of 100 N. The analysis was performed using different distances from the bone level and different thicknesses of cortical bone. The interface area of bone growth was evaluated by analyzing the bone–implant contact (BIC), region of interest (ROI) and total bone area (BAT) parameters obtained through an in vivo histological process and analyzed by scanning electron microscopy (SEM). Bone-level implants were inserted in the rabbit tibiae, with two implants placed per tibia. These parameters were evaluated after three or six weeks of implantation. FEM studies showed that placements 0.5 mm below the bone level presented lower values of stress distribution compared to the other studied placements. The lower levels of mechanical stress were then correlated with the in vivo studies, showing that this position presented the highest BIC value after three or six weeks of implantation. In this placement, vertical bone growth could be observed up the bone level. The smallest thickness of the study showed a better transfer of mechanical loads, which leads to a better osseointegration. In silico and in vivo results both concluded that the implants placed 0.5 mm below the cortical bone and with lower thicknesses presented the best biomechanical and histological behavior in terms of new bone formation, enhanced mechanical stability and optimum osseointegration.

## 1. Introduction

Different factors will affect bone behavior, growth or loss when dental implants are placed at the osseous level. These factors are: macro and micro implant design [1,2,3,4], the separation between placed implants [5], periodontal and bone quality [6], occlusal loading [7], the microgap prone to bacteria colonization in the implant abutment connection and, in consequence, the location of this connection in relation to the bone crest [8,9,10,11,12]. Some studies show peri-implant bone losses of between 1 and 2 mm after the first year of occlusal loading, and from 0.1 to 0.2 mm over successive years [13,14,15]. However, Pellicer et al. observed that different placement of the bone-level implant could produce bone growth with different behavior in an analysis of different clinical studies [16].

Placement of an implant in a deeper position with respect to the bone crest (subcrestal placement) has been suggested as a method that could contribute to maintaining the peri-implant soft and hard tissues in comparison with crestal placement, though this affirmation is subject to controversy. As early as 1969, Branemark [17] recommended placing the implant below the bone crest to prevent implant exposure during bone remodeling.

Different studies [18,19,20] have demonstrated that bone-level dental implants placed approximately 2 mm below the bone crest are associated with significantly less peri-implant bone loss compared to implants placed at crestal level. However, other researchers [20,21,22,23,24,25] have observed greater bone loss with implants placed at subcrestal level. This bone loss has been explained by peri-implantitis [26]; the elimination of biofilm from the dental implants is necessary [27,28,29,30]. In relation to bone growth, the placement of implants in a subcrestal position has been suggested as a method that could contribute to the maintenance of hard and soft peri-implant tissues compared to a crestal placement–though this affirmation is subject to debate. Experimental animal studies [21,25,31,32] and human studies [20,33,34,35] have observed that subcrestal implant placement produces an increase in peri-implant bone loss.

Esposito et al. [36] and Sanz et al. [37] published two meta-analyses that compared efficacy of implant placements in immediate or delayed implant placements. Both studies concluded that more clinical studies are required to establish clear conclusions and clinical guidelines regarding the timing of implant placement. Subsequently, there has been a considerable increase in the number of clinical studies investigating the efficacy of early implant placement. However, there is no systematic review to provide a quantitative and qualitative overview of the recently available evidence on this topic. Hence, there is a need to carry out a study using the finite element method to study the load transfer in different subcrestal, crestal and supracrestal situations. Load transfer is a key factor in bone formation and bone loss [38,39,40]. Tribst et al. [41] worked on the design and analysis of an implant-supported full-arch dental prosthesis with limited occlusal vertical dimension in terms of mechanical improvements by 3D FEA and on posterior dental crowns with functional elasticity gradients [42]. This finite element modeling is validated by rabbit implants in all three positions. In addition, an important factor for load transfer for the same implant design is the thickness of the bone crest. Stress levels in the bone give us the possible bone formation that is validated by the histologies performed. Although bone formation and loss have multiple factors, as described above, this paper aims to clarify the role of occlusal loading.

The cortical bone plays an important role in terms of strain distribution and magnitude in the bone tissue. Ausiello et al. [43] demonstrated different neck designs that can reduce strain values and improve load dissipation in the bone tissue; implants with 10° and 20° neck configuration are preferred instead of straight implant platforms.

The null hypothesis of this research is that the height at which bone-level implants are placed does not matter to osseointegration. Likewise, we hypothesize that the width of the cortical bone does not matter to bone formation around the dental implant.

## 2. Materials and Methods

### 2.1. Finite Element Analysis Methodology

The implant was modeled by using a 3D model discretized using finite elements in order to reproduce the osseointegration response of the implant in both types of bone mandible (cortical and trabecular). Poiate et al. [38] indicated that stress distribution was similar qualitatively in 2D and 3D models but stress magnitude was quite different. It was concluded that 2D models are acceptable when investigating the biomechanical behavior of upper central incisors qualitatively. However, quantitative stress analysis is less reliable in a 2D finite element analysis, because 2D models overestimate the results and do not represent the complex anatomical configuration of dental structures. For these reasons, it was necessary to carry out this biomechanical study on 3D models.

**Figure 1:**
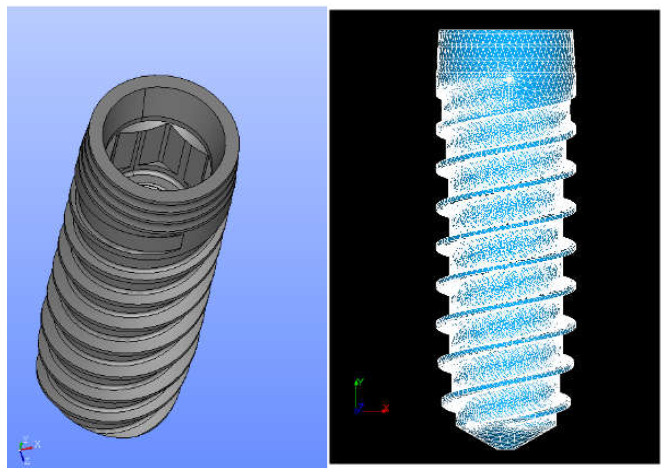
(**Left**) implant model. (**Right**) finite element mesh with 479.132 elements and 106.370 nodes.

The dental implant studied was a bone-level dental implant (Vega, Klockner Dental Implant System, Escaldes-Engordany, Andorra) as can be seen in Figure 1.

**Figure 2:**
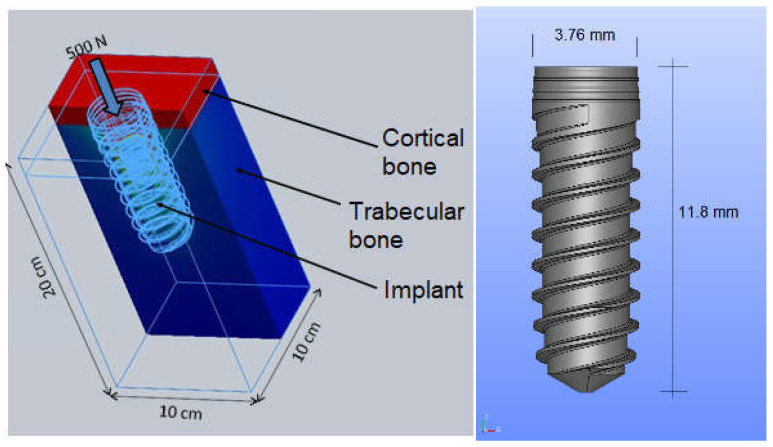
(**Left**) the whole model: implant, cortical bone and trabecular bone. (**Right**) implant.

The preprocessing and finite element meshing of the model were done by using the well-known software tool SolidWorks [44]. The mandible model was set up with an internal-part trabecular bone bounded by a cortical-bone layer of 5 mm thickness. The total height of the mandible model was 35 mm. The combination of all these parts had a height of 45 mm, a bucco-lingual width of 10 mm and a medium-distance length also of 10 mm (see Figure 2). The combined model of implant and bone described herein was shown to be very effective.

The 3D geometry of the implant was provided by the manufacturer as a solid. Then, it was imported into SolidWorks for creating the whole model and further FE analyses. All the materials of the model are elastic and isotropic. Table 1 below [45,46] lists the mechanical properties (Young’s moduli and Poisson’s ratios) [45,46]. The formulation of the FEM and other details on the method are described in several well-known works, among them [45,46].

The geometry of the whole model is displayed in Figure 2.

A sensitivity analysis of the finite element mesh was performed before proceeding with the numerical calculations. As a result of this analysis, the proposed model was meshed using classical linear tetrahedral elements with different refinements: A 3 mm element size for bones and a 0.2 mm element size for all the components of the implant.

Then, the whole model (implant, cortical and trabecular bones) was embedded in all directions. The displacements of the lower and side areas of the model were restrained in such a way as to emulate the real situation of a dental implant inserted into bone (see Figure 2). A finite element analysis (FEA) was then also carried out by using SolidWorks software [44]. The post-processing step, also carried out using SolidWorks, allows the user to obtain all stress distributions, such as Von Mises stress, all over the model. In this model, a perfect contact interface was considered. The compression load applied to the model was 100 N [3,7]. This load was applied as a uniformly distributed pressure on the circular ring at the top of the implant [47,48,49,50,51,52].

### 2.2. Implantation in Animals (In Vivo Analysis)

The in vivo animal analysis was performed using 30 rabbits (New Zealand rabbits): 30 rabbits × 2 tibias = 60 placements for dental implants; 3 placements: subcrestal (−0.5 mm), equicrestal (0 mm), supracrestal (+0.5 mm) × 2 cortical thickness (1.5 mm and 2.5 mm) = 6 possibilities; 6 possibilities × 2 times (3 and 6 weeks) = 12 cases.

Consequently, each condition was tested with 5 dental implants without abutment healing (5 dental implants × 12 cases = 60 tests corresponding 30 rabbits).

This analysis was performed because the Young’s modulus of a rabbit’s tibia is about 21.3 GPa [45,46] which is close to that of the human mandible. The protocol followed for the present research was approved by the University of Córdoba (Spain, register #P01/0144).

After a period of time, between 3 and 6 weeks of implantation, the osseointegration response of the implant was analyzed. It is usually accepted that consolidated bone tissue will appear after 6 weeks of implantation. This is important since the validation of the numerical results was to be done considering consolidated tissue. Sixty samples randomly distributed in rabbits were implanted in the proximal tibiae of the animals (2 samples in each tibia), then twenty-eight were used for the 1st implantation period and the other twenty samples for the 2nd implantation period.

Once the proposed time points were reached, the animals were sacrificed and samples were collected. Non-decalcified samples were then fixed in a formaldehyde solution at 10% in order to keep the tissue structure intact.

The collected tissues and samples were cut with a diamond saw (Exakt 310, Exakt, Norderstedt, Germany) to reduce their size. To ensure good bone tissue fixation, the cut tissues were then immersed in a formaldehyde solution for forty-eight hours. Next, the samples were dehydrated by immersing them in ethanol solutions. Once it was observed that the samples were completely dehydrated, they were embedded in a resin of Technovit-type methyl methacrylate (Kulzer-Heraus, Hanau, Germany). To improve resin penetration, the resin’s concentration was continuously increased with time and using a 50 rpm-constant stirring under vacuum. The tissues inside the resin were then photo-polymerized (using a Histolux light control unit, Kulzer-Heraus, Hanau, Germany) and externally cooled with water. Finally, the samples were placed under white and ultraviolet light to get a transparent solid brick that could be cut.

The brick of polymerized resin was cut into 4 mm-thick slices by using a diamond saw (Exakt 310, Exakt, Norderstedt, Germany). The samples were polished with abrasive papers until they exhibited a flat surface that allowed optimal visualization (Exakt 400 CS polishing machine, Exakt, Germany). The samples were finally covered with carbon and analyzed with the SEM.

Polished specimens were individually analyzed by employing a surface-scanning electron focused ion beam system (Zeiss, Jena, Germany) with backscattered electron detector to visualize non-decalcified bone tissue. All the observations were carried out at a potential charge of 15 kV and an 8 mm-working distance to obtain a resolution down to 1.1 nm. The SEM images were merged using the software ImageJ (NIH, Bethesda, MD, USA) to get a single image of high resolution.

These images were used to evaluate the bone–implant contact (BIC) parameter, which relates the percentage of mineralized tissue in close contact with the implant’s surface. The BIC parameter was evaluated at 20 magnifications from the implant’s perimeter along neck length for all the samples. Additionally, bone ingrowth into the threads (BAT) and bone density at 1 mm outside the implant threads (ROI) were quantified by using the histologies. More than one thousand samples were analyzed with the optical microscope.

The data were analyzed with a single-factor ANOVA and the Kruskal–Wallis (non-parametric) test in order to verify if there were important differences between experimental groups. For this purpose, Minitab16 software (Minitab Inc., State College, PA, USA) was used, assigning a 95% confidence interval.

## 3. Results and Discussion

The described model was analyzed using the finite element method (FE) in the three positions (cases) described:

- Case A: 0.5 mm above the bone surface (h = +0.5).
- Case B: at the same level of the bone surface (h = 0).
- Case C: 0.5 mm below the bone surface (h = −0.5).

Since the stability of the implant can vary according to cortical-bone thickness, the three cases mentioned above were analyzed for two different thicknesses of the cortical bone:

t = 1.5 mm (cortical thickness).
t = 2.5 mm (cortical thickness).

The results of the FE analyses in all the models are reported below.

**Figure 3:**
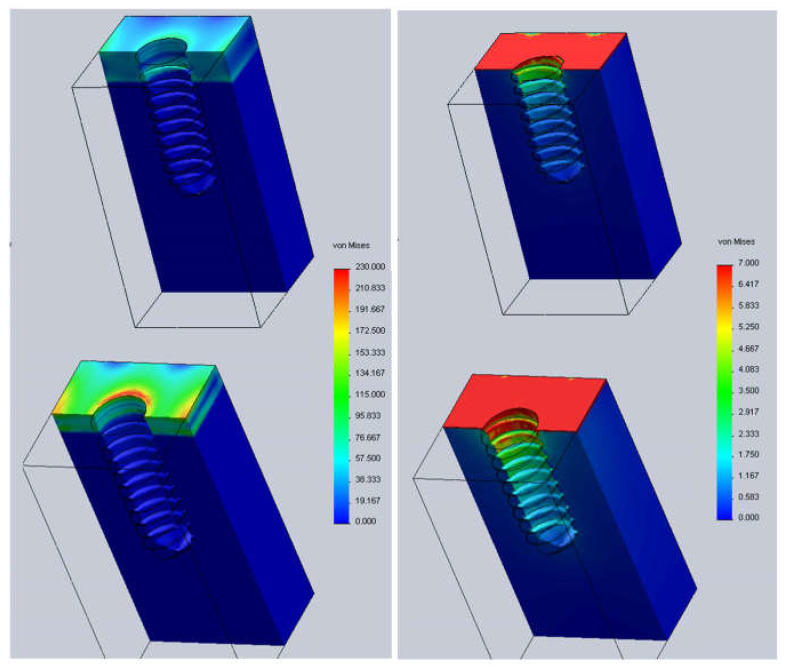
Von Mises stresses (MPa). **Left** column: cortical bone. **Right** column: trabecular bone. **Top** row: cortical thickness 2.5 mm. **Bottom** row: cortical thickness 1.5 mm.

Figure 3 shows the Von Mises stresses in the implant at position h = 0; that is, the implant is positioned flush with the bone’s level. Stresses are much greater in cortical bone (over 230 MPa) than in trabecular bone (7 MPa). In cortical bone, stresses show a maximum value at the implant surface. However, in trabecular bone, the stresses are at maximum in a region close to the head of the implant and they decrease progressively with bone depth. When the thickness of the cortical bone decreases, the stresses on both the cortical and the trabecular bone increase.

**Figure 4:**
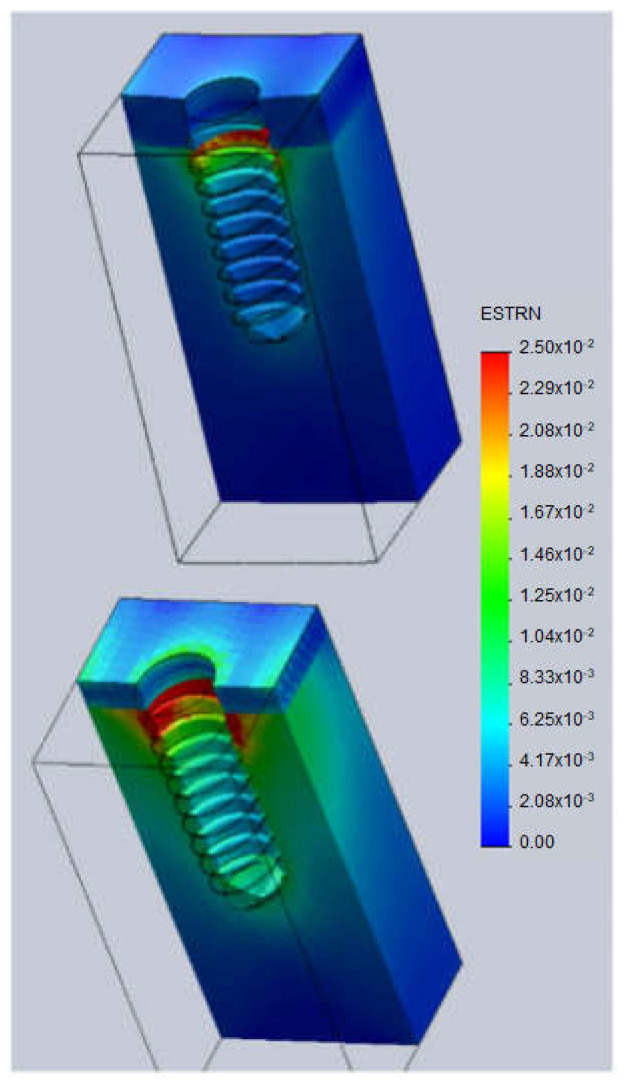
Strains (mm/mm). **Top**: cortical thickness 2.5 mm. **Bottom**: cortical thickness 1.5 mm.

Figure 4 shows the strains computed in the implant modeled at position h = 0. Unlike the stresses distribution, the strains are greater in the trabecular bone (over 2.5%) than in the cortical bone where strains are below the 0.6% regardless of the cortical bone thickness. In the trabecular bone, larger strains are observed in the region close to the cortical bone and around the top surface of the implant. As the cortical bone thickness decreases, the strains in both cortical and trabecular bone increase.

**Figure 5:**
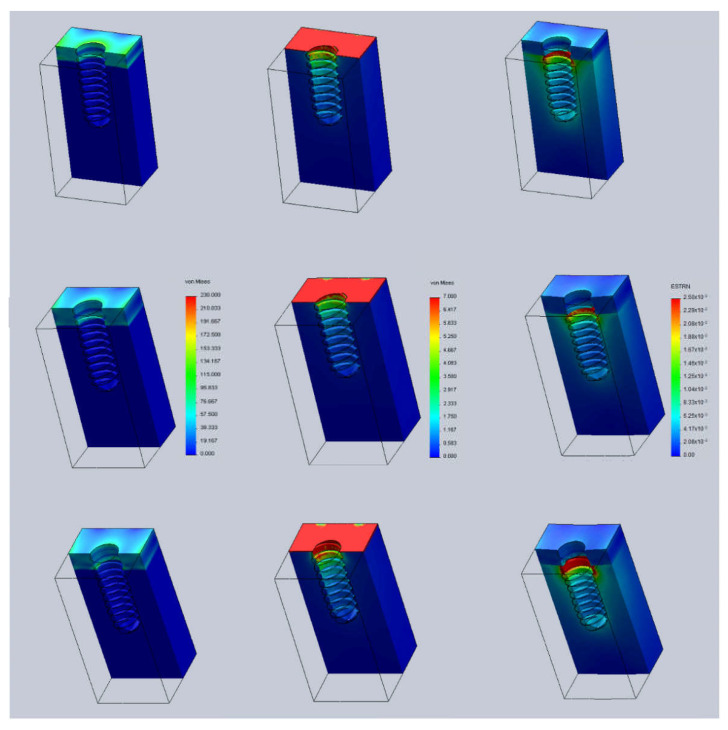
Von Mises stresses (MPa) and strains (mm/mm) with a cortical-bone thickness of 2.5 mm. **Left** column: stresses at cortical bone. **Center** column: stresses at trabecular bone. **Right** column: strains. **Top** row: Case A (h = +0.5). **Middle** row: Case B (h = 0). **Bottom** row: Case C (h = −0.5).

Figure 5 displays both stresses and strains with a cortical-bone thickness of 2.5 mm in the three cases studied herein. It can be observed that as the implant penetrates further into the jaw, the stresses on the cortical bone (left column of figure) decrease by around 100 to 50 MPa. Regarding the strains (right column of figure), changes in cortical bone strains are negligible, but in the trabecular bone, the region where the strain exceeds 2.5% increases as implantation depth increases.

**Figure 6:**
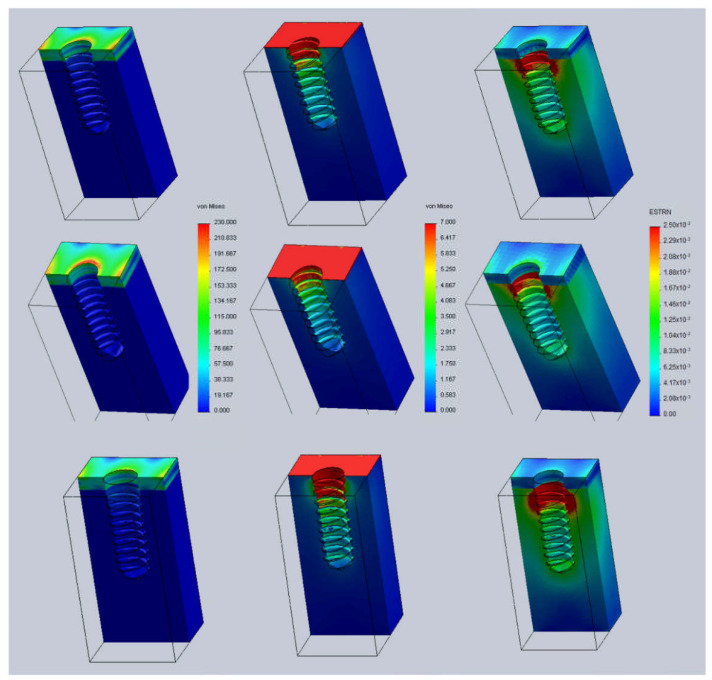
Von Mises stresses and strains with a cortical-bone thickness of 1.5 mm. **Left** column: stresses at cortical bone. **Center** column: stresses at trabecular bone. **Right** column: strains. **Top** row: Case A (h = +0.5). **Middle** row: Case B (h = 0). **Bottom** row: Case C (h = −0.5).

The Figure 6 shows similar results as in Figure 6 but now the cortical-bone thickness is smaller (1.5 mm).

Similar to the case of the thicker cortical bone, it can be seen that as the implant penetrates further into the jaw, the stresses on the cortical bone (left column of figure) decrease, whereas stresses on the trabecular bone (center column of figure) increase. However, unlike the case of the thicker cortical bone, both the stresses in the cortical and trabecular bone are greater, and the difference when the implant is buried further in the bone is more evident. The maximum stresses in the cortical bone decrease by around 230 to 160 MPa, whereas in the trabecular bone, the area where the stresses are greater than 7 MPa increases by up to three threads of the implant thread. Changes in the strain of the cortical bone in the case of the thinnest cortical bone are still negligible (right column figures), but in the trabecular bone, the region where the strain exceeds 2.5% is sensitive to implant position.

**Figure 7:**
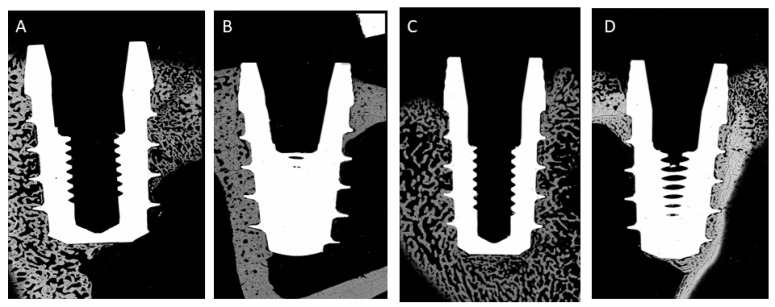
Histologies of the different bone-level dental implants inserted 0.5 mm above of bone level (h = +0.5 mm). (**A**) 3 weeks implanted and bone thickness of 1.5 mm; (**B**) 6 weeks implanted and bone thickness of 1.5 mm; (**C**) 3 weeks implanted and bone thickness of 2.5 mm; (**D**) 6 weeks implanted and bone thickness of 2.5 mm.

**Figure 8:**
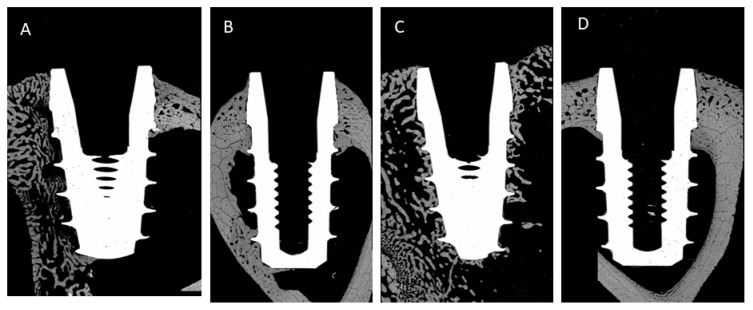
Histologies of the different bone-level dental implants inserted at bone level (h = 0 mm). (**A**) 3 weeks implanted and bone thickness of 1.5 mm; (**B**) 6 weeks implanted and bone thickness of 1.5 mm; (**C**) 3 weeks implanted and bone thickness of 2.5 mm; (**D**) 6 weeks implanted and bone thickness of 2.5 mm.

**Figure 9:**
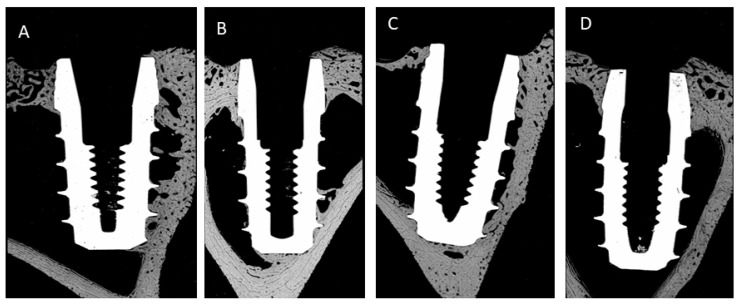
Histologies of the different bone-level dental implants inserted at 0.5 below the bone level (h = −0.5 mm). (**A**) 3 weeks implanted and bone thickness of 1.5 mm; (**B**) 6 weeks implanted and bone thickness of 1.5 mm; (**C**) 3 weeks implanted and bone thickness of 2.5 mm; (**D**) 6 weeks implanted and bone thickness of 2.5 mm.

Figure 7, Figure 8 and Figure 9 show some histologies of the bone-level dental implants for those placed 0.5 mm above the bone, at the same level as the bone and 0.5 mm below the bone level, at the different implantation times: 3 or 6 weeks, and for different bone thicknesses: 1.5 and 2.5 mm, respectively.

The high-sensitivity image analysis studies performed in the scanning electron microscope for each dental implant allowed us to obtain the results of bone growth: BIC, BAT and ROI, which indicate the osseointegration capacity of the dental implants under the different conditions studied. The results are shown in Table 2 for those implanted in bone with a cortex thickness of 1.5 mm and in Table 3 for those with a cortex thickness of 2.5 mm.

In all cases with statistically significant differences (*p* < 0.05), osseointegration levels (BIC, BAT and ROI) are greater at the implantation time of 6 weeks than at 3 weeks, as expected.

The results for the two cortical thicknesses studied show that dental implants implanted 0.5 mm below bone level give the highest levels of osseointegration compared to implants implanted at bone level and those implanted above bone level (0.5 mm). These differences were statistically significant.

These results validate the finite element model applied since we could observe that it was in this condition where the transfer of mechanical loads to the cancellous tissue was greater. It should be borne in mind that load transfer in this type of bone tissue favors the formation of new bone, as cancellous tissue is much more vascularized than cortical tissue. It can also be observed that dental implants placed at bone level have better levels of osseointegration, but with a non-statistically significant difference, with *p* < 0.05 (*p* = 0.13). Dental implants placed above the bone level have the worst BIC, BAT and ROI results as established by the load transfer results obtained by finite element analysis.

From the results shown in Table 2 and Table 3, it can also be affirmed that the thickness of the cortical bone exerts an opposite effect on osseointegration, i.e., the thicker the cortical bone, the lower the BIC, BAT and ROI values compared to the values obtained for implants placed in cortical bones of lesser thickness. These results have been obtained in different clinical studies but previously there existed no clear interpretation of the reason behind them. The finite elements have helped us understand that load transfer when thickness is 1.5 mm is much more effective for bone growth than when thickness is 2.5 mm, as in this case, far greater tension is absorbed and produces a significant decrease in the level of tension in the trabecular bone, which does not favor the formation of new bone. As previously described, cancellous bone favors the formation of new bone with mechanical stimulation due to the fact that it is more vascularized than compact bone.

This research was carried out using tapered bone-level dental implants with a certain neck design. It should be noted that although commercial dental implants are very similar in shape for optimizing mechanical transfer to the bone, different designs could modify some of the results of those studied in this work. This limitation would not affect the thickness of the cortex, but there could be small differences in the angles of conicity of the implant. It must be taken into account that the designs of the implant necks cannot be very angulated, since it has been observed that mechanical stress on the cortical bone causes so much of a compressive load that the blood vessels collapse, producing bone necrosis. This is why, in this type of dental implant, large bone resorption occurs, causing empty areas of V-shaped bone that are susceptible to bacterial colonization to initiate the peri-implantitis process.

The results obtained in silico and validated in vivo are in agreement with a meta-analysis realized by Bassir et al. [53]. In this study, there was no statistically significant difference in crestal bone changes between subcrestal and equicrestal implant positioning; however, subcrestal position resulted in higher bone levels. Neither mucosal recession nor vertical mucosa thickness were influenced by different implant placement depths. A 5-year randomized clinical trial by de Siqueira et al. [54] showed that subcrestal implant placement had less bone loss and resulted in no implant thread exposure, whereas with equicrestal placement, thread exposure occurred in one implant after a 5-year follow-up. It is therefore speculated that a subcrestal implant placement of at least 1 mm can prevent possible biological complications due to implant rough surface exposure. Our results are also in agreement with previous studies that demonstrated that subcrestal implants produce more bone tissue and minimize bone resorption [45,46,47,48,49,50,51,52,53,54,55,56,57,58,59,60,61,62,63,64]. In addition, the clinical relevance of this result is that subcrestal placement may reduce the risk of having peri-implantitis by minimizing rough surface exposure [62,63]. A similar finding was reported by Vervaeke and coworkers in a 2-year follow-up study [64] and in a 3-year follow-up using platform-switched implants [57].

As we have seen in this contribution, the finite element simulation and the validation of the in vivo tests were well correlated. However, there are limitations to this study, as it is valid for only one dental implant design. Although from a macroscopic point of view there are few differences in bone-level implant designs, differences in the neck and body taper could cause some variations. Moreover, the load applied was static; thus, dynamic study would be beneficial, although the complexity of cyclic tooth movement makes this a very difficult simulation at this time. This research served to negate the null hypothesis we made in the introduction. As we were able to demonstrate, the location where the dental implant is placed and the thickness of the bone crest have an important influence on osseointegration. Undoubtedly, new technologies, such as the use of magnetic technologies in implantology [65], or new simulation systems with dynamic diffusion elements [66] could improve analysis in the future. A limitation of this study we also have to consider is that the dental implants did not have prostheses; sometimes, loads are modified by the types of prostheses changing the levels of bone growth, as studied by Tribst et al. [67]. However, the in vivo results of this study simulate mechanical loads, as the dental implants were placed in the tibiae of the rabbits, which were cracked due to the mobility of the rabbits inside the cages. Technicians from the Faculty of Veterinary Sciences caused them to move for feeding and drinking for at least 125 min each day.

Despite these limitations, the study performed is in line with the results of published clinical studies and therefore the model used both in silico and in vivo is adequate [68,69,70,71,72].

## 4. Conclusions

FEM simulation studies showed that bone-level dental implants placed subcrestally had a lower load transfer to the bone than dental implants at bone level and above the bone crest. Bone formation parameters BIC, BAT and ROI were higher for implants placed subcrestally (0.5 mm) at 3 or 6 weeks after implantation. Bone crest values also influenced bone formation, with 1.5 mm thickness giving better results than 2.5 mm thickness. The occlusal load transfer favored bone formation, followed by equicrestal dental implants, whereas the worst load transfer was observed in implants placed above the bone crest. It was also determined that the thinnest cortical bone favored adequate load transfer and that the more vascularized cancellous tissue favored bone growth. The simulation in silico results confirmed the results of the in vivo studies, where the best load transfer and strain was observed in 0.5 mm subcrestal placement. In addition, the importance of the thickness of cortical bone was validated by means of histologies. These showed that smaller thicknesses favor bone formation.

## Figures and Tables

**Table 1:** Mechanical properties of all the model’s materials.

Material	Young’s Modulus (MPa)	Poisson’s Ratio
Implant	110,000	0.34
Cortical bone	19,400	0.30
Trabecular bone	5600	0.28

**Table 2:** Bone index contact (BIC), total bone area (BAT) and region of interest bone growth (ROI) for different types of Ti dental implant surfaces after 3 or 6 weeks of implantation. Cortical thickness of 1.5 mm. Statistical differences for each column are indicated by single asterisk (*p* < 0.05). Thickness: 1.5 mm.

BIC	3 Weeks	6 Weeks
Above 0.5 mm	22% ± 5%	27% ± 8%
Bone level	29% ± 6%	33% ± 9%
Below 0.5 mm	35% ± 6% *	55% ± 8% *
BAT	3 Weeks	6 Weeks
Above 0.5 mm	26% ± 4%	32% ± 7%
Bone level	32% ± 9%	40% ± 8%
Below 0.5 mm	40% ± 6% *	62% ± 5% *
ROI	3 Weeks	6 Weeks
Above 0.5 mm	18 ± 7%	25% ± 6%
Bone level	24% ± 6%	38% ± 8%
Below 0.5 mm	30% ± 5% *	55% ± 7% *

**Table 3:** Bone index contact (BIC), total bone area (BAT) and region of interest bone growth (ROI) for different types of Ti dental implant surfaces after 3 or 6 weeks of implantation. Cortical thickness of 1.5 mm. Statistical differences for each column are indicated by single asterisk (*p* < 0.05). Thickness: 2.5 mm.

BIC	3 Weeks	6 Weeks
Above 0.5 mm	12% ± 3%	19% ± 5%
Bone level	18% ± 6%	25% ± 9%
Below 0.5 mm	29% ± 4% *	39% ± 9% *
BAT	3 Weeks	6 Weeks
Above 0.5 mm	16% ± 4%	27% ± 8%
Bone level	26% ± 6%	35% ± 9%
Below 0.5 mm	35% ± 5% *	48% ± 5% *
ROI	3 Weeks	6 Weeks
Above 0.5 mm	20 ± 5%	26% ±4%
Bone level	25% ± 6%	32% ± 5%
Below 0.5 mm	29% ± 8% *	45% ± 8% *

## Data Availability

The data presented in this study are available on request from the corresponding author. The data are not publicly available due to some confidential results.

## Abbreviations

BAT	Total bone area
BIC	Bone–implant contact
FEA	Finite element analysis
FEM	Finite element method
GPa	GigaPascals
h	Height
mm	Millimeter
MPa	MegaPascals
ROI	Region of interest
SEM	Scanning electron microscopy
3D	Three-dimensional
